# Benefit of B7-1 staining and abatacept for treatment-resistant post-transplant focal segmental glomerulosclerosis in a predominantly pediatric cohort: time for a reappraisal

**DOI:** 10.1007/s00467-022-05549-7

**Published:** 2022-05-04

**Authors:** George W. Burke, Jayanthi Chandar, Junichiro Sageshima, Mariella Ortigosa-Goggins, Pooja Amarapurkar, Alla Mitrofanova, Marissa J. Defreitas, Chryso P. Katsoufis, Wacharee Seeherunvong, Alexandra Centeno, Javier Pagan, Lumen A. Mendez-Castaner, Adela D. Mattiazzi, Warren L. Kupin, Giselle Guerra, Linda J. Chen, Mahmoud Morsi, Jose M. G. Figueiro, Rodrigo Vianna, Carolyn L. Abitbol, David Roth, Alessia Fornoni, Phillip Ruiz, Gaetano Ciancio, Eduardo H. Garin

**Affiliations:** 1 Division of Kidney-Pancreas Transplantation, Department of Surgery, Miami Transplant Institute, University of Miami Miller School of Medicine, 1801 NW 9th Ave, Highland Professional Building, Miami, FL 33136 USA; 2 Division of Pediatric Kidney Transplantation, Department of Pediatrics, Miami Transplant Institute, University of Miami Miller School of Medicine, Miami, FL 33136 USA; 3 Division of Transplant Surgery, Department of Surgery, University of California Davis School of Medicine, Sacramento, CA 95817 USA; 4 Katz Family Division of Nephrology and Hypertension, Department of Medicine, and the Miami Transplant Institute, University of Miami Miller School of Medicine, Miami, FL 33136 USA; 5 Division of Nephrology, Department of Medicine, Emory University School of Medicine, Atlanta, GA 30309 USA; 6 Research, Katz Family Division of Nephrology and Hypertension, Department of Medicine, University of Miami Miller School of Medicine, Miami, FL 33136 USA; 7 Division of Pediatric Nephrology, Department of Pediatrics, University of Miami Miller School of Medicine, Miami, FL 33136 USA; 8 Transplant Clinical Pharmacy Services, Miami Transplant Institute, Jackson Memorial Hospital, Miami, FL 33136 USA; 9 Division of Liver and GI Transplantation, Department of Surgery, Miami Transplant Institute, University of Miami Miller School of Medicine, Miami, FL 33136 USA; 10 Katz Family Division of Nephrology and Hypertension, Department of Medicine, University of Miami Miller School of Medicine, Miami, FL 33136 USA; 11 Transplant Pathology, Department of Surgery, Miami Transplant Institute, University of Miami Miller School of Medicine, Miami, FL 33136 USA; 12 Division of Nephrology, Department of Pediatrics, University of Florida School of Medicine, Gainesville, FL 32610 USA

**Keywords:** Podocyte, Proteinuria, Nephrotic syndrome, Focal segmental glomerulosclerosis, Kidney transplantation, B7-1, Abatacept

## Abstract

**Background:**

Primary FSGS manifests with nephrotic syndrome and may recur following KT. Failure to respond to conventional therapy after recurrence results in poor outcomes. Evaluation of podocyte B7-1 expression and treatment with abatacept (a B7-1 antagonist) has shown promise but remains controversial.

**Methods:**

From 2012 to 2020, twelve patients developed post-KT FSGS with nephrotic range proteinuria, failed conventional therapy, and were treated with abatacept. Nine/twelve (< 21 years old) experienced recurrent FSGS; three adults developed de novo FSGS, occurring from immediately, up to 8 years after KT. KT biopsies were stained for B7-1.

**Results:**

Nine KTRs (75%) responded to abatacept. Seven of nine KTRs were B7-1 positive and responded with improvement/resolution of proteinuria. Two patients with rFSGS without biopsies resolved proteinuria after abatacept. Pre-treatment UPCR was 27.0 ± 20.4 (median 13, range 8–56); follow-up UPCR was 0.8 ± 1.3 (median 0.2, range 0.07–3.9, *p* < 0.004). Two patients who were B7-1 negative on multiple KT biopsies did not respond to abatacept and lost graft function. One patient developed proteinuria while receiving belatacept, stained B7-1 positive, but did not respond to abatacept.

**Conclusions:**

Podocyte B7-1 staining in biopsies of KTRs with post-transplant FSGS identifies a subset of patients who may benefit from abatacept.

**Graphical abstract:**

A higher resolution version of the Graphical abstract is available as Supplementary information

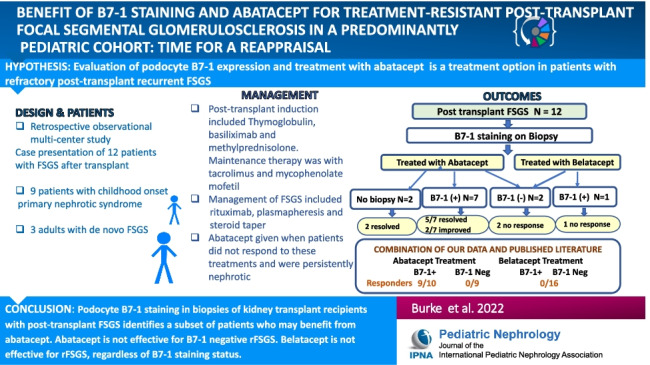

**Supplementary Information:**

The online version contains a graphical abstract available at 10.1007/s00467-022-05549-7.

## Introduction

Focal segmental glomerulosclerosis (FSGS) is a primary glomerular disorder that manifests with nephrotic syndrome (NS) [1]. Progression to kidney failure occurs in 40–60% of patients within 10–20 years of diagnosis [2], making this the most common primary glomerular disease leading to kidney failure in the USA. Following transplantation, recurrent FSGS (rFSGS) occurs in 30–40% of adults [3] and up to 80% in high-risk pediatric patients [4]. Risk factors for rFSGS include developing steroid resistance at younger age (less than 6 years old), Caucasian race, rapid progression to kidney failure (less than 3 years from diagnosis), severe proteinuria immediately prior to transplantation, two renal risk alleles for ApoL1 [5], and the loss of previous allograft(s) to recurrence [2, 6–8]. Recurrent FSGS increases the risk of kidney dysfunction and early graft loss [9, 10].

Treatment of rFSGS has included steroids, plasmapheresis, calcineurin inhibitors, and rituximab [7, 11, 12]. Successful treatment of rFSGS results in 100% 5-year graft survival rate, whereas, failure to effectively treat rFSGS results in a dismal (36.5%) 5-year graft survival rate [13].

We have previously reported that the peri-operative use of rituximab led to the reduction of recurrent proteinuria in high-risk pediatric kidney transplant (KT) recipients with FSGS [14], providing evidence that this was mediated by an effect on the podocyte. For those KTRs who experienced recurrent proteinuria, despite receiving peri-operative rituximab, our team has pursued a podocyte-directed approach [14–23]. In 2004, podocyte expression of B7-1, a co-stimulatory molecule on antigen-presenting cells [24], was found to be associated with the development of nephrotic syndrome in patients and experimental animals [25]. Therefore, we reasoned that interference with B7-1 expression may exert a podocyte-protective effect to reduce the degree of proteinuria in KTRs with rFSGS.

In 2013, we demonstrated the induction of podocyte B7-1 expression after exposure of normal donor kidneys to the circulation of patients with FSGS. Specifically, we performed pre- and post-reperfusion KT biopsies in the operating room in KTRs who were at risk for rFSGS, and stained them for B7-1 and the podocyte-colocalizing marker synaptopodin. We identified B7-1 on podocytes from post-, but not pre-reperfusion biopsies of KT recipients who experienced early recurrent proteinuria [26] and used abatacept, which binds to B7-1 [27], as a potential therapeutic agent. Abatacept treatment within the first week of KT resulted in improvement or resolution of proteinuria in 4 KT recipients with rFSGS [26].

After reporting our experience [26], others were unable to identify podocyte B7-1 on KT biopsies [28–31], and did not find abatacept, or other CTLA4Igs, specifically belatacept, effective in treating rFSGS [28, 32, 33]. This update of our experience suggests there may be a subset of patients with post-transplant FSGS who express B7-1 on podocytes and respond to abatacept.

## Materials and methods

**Fig. 1:**
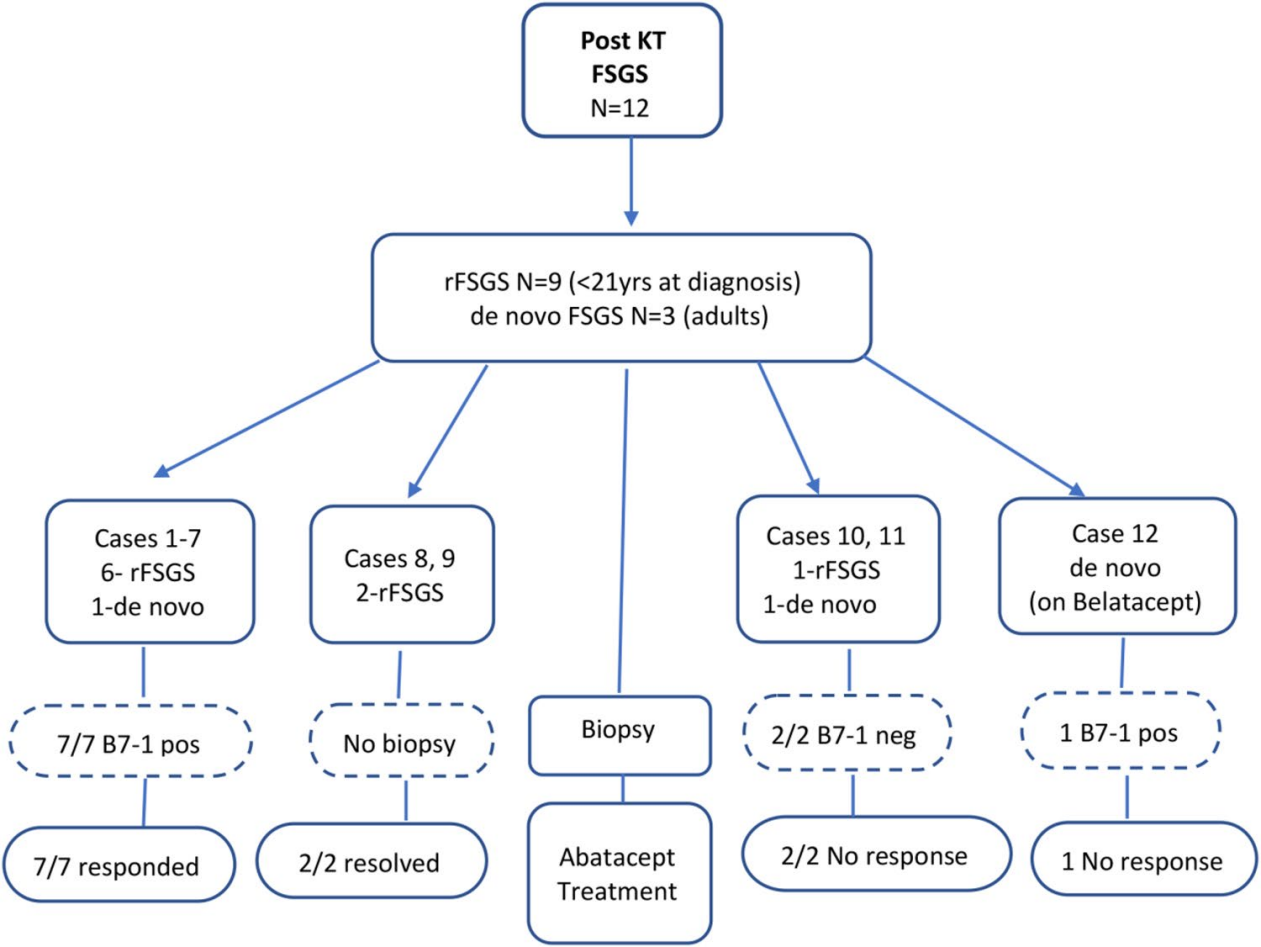
Flow diagram of twelve post-transplant FSGS patients showing age (pediatric or adult), recurrent FSGS vs. de novo FSGS, B7-1 KT biopsy staining results, and response to abatacept

We report our multi-center (University of Miami Miller School of Medicine and University of Florida School of Medicine) experience with twelve KTRs who developed proteinuria after transplantation, from January 2012 to April 2020. Nine pediatric patients (age 2–20 years at diagnosis) were diagnosed with primary FSGS prior to KT. These nine patients were screened for genetic causes (mutations in podocyte proteins, e.g., nephrin and podocin) whenever possible, as well as for infections (HIV, CMV, EBV, etc.), drugs and toxins, maladaptive disorders (reduced nephron number), and metabolic issues such as obesity and diabetes [34]. Three patients (adults, age 34, 56, and 78 years at diagnosis) developed biopsy-proven de novo FSGS (see Fig. 1 and Table 1). Five KTRs were AA, four were Caucasian, and three were Hispanic.

**Table 1:** Clinical characteristics of 12 patients with post-KT FSGS

Case^1^	Patients			Biopsy findings					Treatment					Response^10^
	Age at primary diagnosis (yrs)	Gender^2^/race^3^	Donor type	Biopsy	H&E^5^	EM^6^ (FPE)	B7-1 (intensity) (0–3 +)	Peak UPCR^7^	Abatacept (IV/SC)/(doses)	PP^8^ (total sessions)	Ritux^9^ (doses)	ACEI/ARB	Other treatment	
**I. B7-1 positive cases that responded to abatacept**														
1	2	M/AA	DD	Yes	FSGS	Mod	Pos (1 +)	13	Yes (IV, 2)	5	Yes (1)	Yes		Resolution
2	8	M/C	LRD	Yes	FSGS	Yes (NA)	Pos (NA)	53	Yes (IV, 1)	7	No	NA	Cyclophosphamide	Resolution [36]
3^1^	34	F/AA	DD	Yes	FSGS	Min	Pos (2 +)	8	Yes (SC, 4)	3	Yes (1)	No		Resolution
4	6	M/H	DD	Yes	AMR/FSGS	Mod	Pos (2 +)	9	Yes (IV, 1)	4	Yes (1)	Yes		Resolution
5	8	M/AA	DD	Yes	FSGS	Mild	Pos (2 +)	12	Yes (SC, 4)	8	Yes (1)	Yes		Improvement
6	13	M/C	LRD(M)	Yes	FSGS	Mod	Pos (3 +)	56	Yes (SC/IV, 12/8)	> 40	Yes (2)	Yes		Improvement
7	4	M/H	LRD(M)	Prior^4^	–	–	–	10	Yes (IV, 4)	No	No	Yes	Steroids	Resolution
**II. Cases without KT biopsies that responded to abatacept**														
8	2	M/H	DD	No	–	–	–	44	Yes (IV, 4)	No	No	No		Resolution
9	5	M/AA	DD	No	–	–	–	38	Yes (IV, 3)	6	Yes (2)	Yes		Resolution
**III. B7-1 negative cases that did not respond to abatacept**														
10^1^	56	F/AA	DD	Yes × 3	FSGS	Mild	Neg × 3	50	Yes (SC, 4)	10	Yes (1)			No resolution/graft loss
11	20	M/C	LRD(M)	Yes × 3	FSGS	Mod	Neg × 3	28	Yes (IV, 4)	> 40	Yes (2)	Yes	Belatacept/IVIg	No resolution/graft loss
**IV. B7-1 positive case that received belatacept and did not respond to abatacept treatment**														
12^1^	78	F/C	DD	Yes	FSGS	Mod	Pos (2 +)	16	Yes (IV, 4)	11	Yes (1)	Yes	Belatacept	No resolution/graft loss

^1^Case—de novo

^2^* M*, male; *F*, female

^3^*AA*, African American; *C*, Caucasian; *H*, Hispanic

^4^Prior post-reperfusion biopsy (see case report 7)

^5^Hemotoxylin and eosin (light microscopy): *MTI*, minimal tubulo-interstitial injury; *AMR*, antibody-mediated rejection

^6^Electron microscopic finding of foot process effacement (FPE): *NA*, not available; degree, none, minimal, mild, moderate

^7^*UPCR*, urine protein/creatinine ratio

^8^Plasmapheresis (number of session)

^9^Rituximab 375 mg/m^2^, number of doses

^10^Resolution = UPCR < 0.2; improvement: see text

**Fig. 2:**
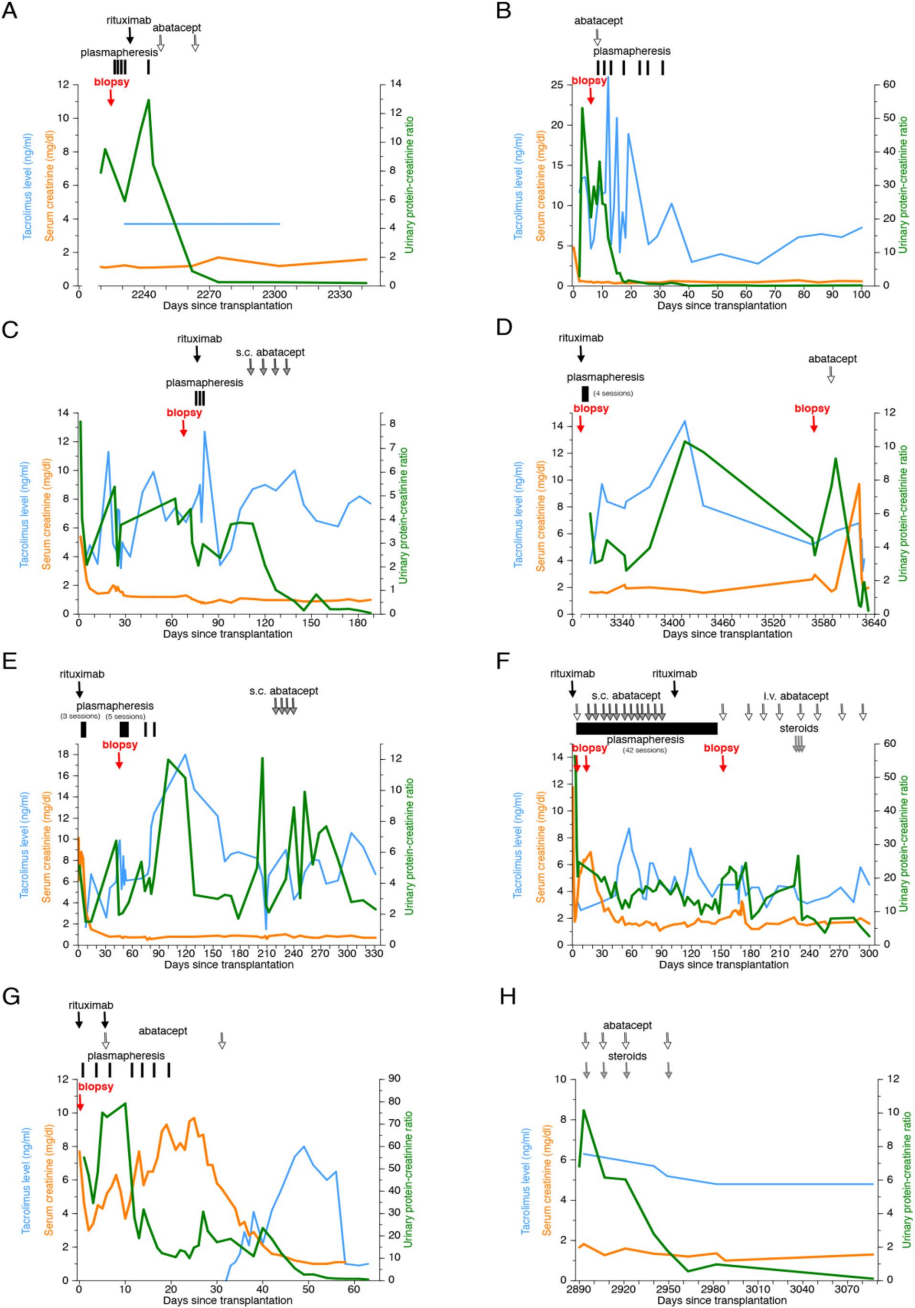
B7-1 positive cases of KT recipients with recurrent FSGS that responded to abatacept. Each graph shows post-transplant serum creatinine levels, tacrolimus levels, and urine protein/creatinine ratio. **A**–**H** Cases 1–7. Also shown: rituximab infusion time(s), plasmapheresis episodes, steroids, and abatacept treatment

These KTRs received induction therapy including thymoglobulin (3–5 doses, 1 mg/kg), basiliximab (2 doses, 10–20 mg/dose) [35], one dose of rituximab (375 mg/m^2^) given peri-operatively [14], and steroids, unless otherwise stated. Maintenance immunosuppression included tacrolimus and mycophenolate mofetil. Steroids were continued at the discretion of the treating physician. All patients experienced nephrotic range proteinuria, defined as UPCR > 3.5 g/g (left as a numerical value only throughout the text), after KT (see Table 1). The clinical course graphs (Figs. 2, 4, 6, 7) include tacrolimus levels or calcineurin inhibitor treatment periods, serum creatinine, and UPCRs. For those KTRs who underwent a KT biopsy (*n* = 10), light microscopy (H&E) and electron microscopy (EM) results are included, and B7-1 stains were obtained. The B7-1 staining results are presented semiquantitatively and expressed based on the degree of intensity, 0 to 3 + . Abatacept was administered intravenously (IV) (10 mg/kg/dose; *n* = 9) or subcutaneously (SC) (250 mg/dose, times four doses; *n* = 3). One patient received both SC and IV abatacept treatment. The total number of doses and route of administration, either IV or SC, were determined by the treating physician. This study was approved by the Institutional Review Board of the University of Miami Miller School of Medicine and the University of Florida School of Medicine.

### Anti-hB7-1 immunohistochemistry (IHC) protocol for kidney transplant biopsy samples

Fresh kidney biopsies were fixated in 10% neutral buffered formalin for 1 h at room temperature. The biopsies were then placed in the Leica PELORIS automated processor (Leica Microsystems—headquarters: Buffalo Grove, IL) to begin processing in 10% neutral buffered formalin for 1 min (at ambient temperature and pressure). To remove residual 10% neutral buffered formalin and begin dehydration, the biopsies were next immersed in 85% ethanol for 1 min (at ambient temperature and pressure), then drained and replaced with another 85% ethanol for 6 min (at 55 °C and ambient pressure). The biopsies were then immersed in an 80% ethanol/20% isopropyl alcohol solution for 1 min (at ambient temperature and pressure), then drained and replaced with another 80% ethanol/20% isopropyl alcohol solution for 6 min (at 55 °C and ambient pressure). To remove residual ethanol and prepare the biopsies to transition into paraffin, the biopsies were immersed in isopropyl alcohol for 1 min (at ambient temperature and pressure), drained and replaced with another isopropyl alcohol for 1 min (at ambient temperature and pressure), and again drained and replaced with another isopropyl alcohol for 12 min (at ambient temperature and pressure). To remove the isopropyl alcohol from the biopsies and begin infiltrating with the paraffin embedding media, the biopsies were immersed in paraffin wax for 20 min (at 85 °C in vacuum). The paraffin was then drained and replaced with paraffin wax for 5 min (at 85 °C in vacuum), and again drained and replaced with paraffin wax for 1 min (at 85 °C in vacuum). The processed biopsies were then embedded in fresh paraffin wax, cooled, and sectioned at 3 µm. Lymph node was used as the control.

The slides were placed in the Leica Immunohistochemistry Autostainer BOND III and deparaffinized in the oven for 30 min, which was followed by Bond Dewax solution for 30 min at 72 °C, Bond Dewax solution (2 changes), 100% reagent alcohol (3 changes, 30 s each), 1 X concentration Bond Wash solution (2 changes), and 1 X concentration Bond Wash solution (1 change for 5 min). While in the Leica Immunohistochemistry Autostainer BOND III the slides were pre-treated with Heat Induce Epitope Retrieval solution 2 (which contains an EDTA-based buffer and surfactant) at a pH of 8.9–9.1 at 100 °C for 20 min, then cooled to room temperature for 12 min. The sections were then washed at room temperature with 1 X concentration Bond Wash solution (Leica, Cat #: AR9590; contains tris-buffered saline, surfactant and 3.5% ProClin 950. pH 7.5–7.7) and incubated with the primary antibody Anti-hB7-1 (R&D Systems, parent company: Bio-techne—headquarters: Minneapolis, MN) purified mouse monoclonal IgG1 (clone: 37,711, dilution: 1:20) for 15 min at room temperature. The sections were washed with 1 X concentration Bond Wash solution (3 changes) at room temperature, and incubated at room temperature for 8 min with the post-primary (polymer penetration enhancer containing 10% animal serum in tris-buffered saline and 0.09% ProClin 950). The sections were washed with 1 X concentration Bond Wash solution (3 changes, 2 min each) at room temperature. The slides were incubated at room temperature for 8 min with polymer poly-HRP anti-mouse/rabbit IgG (contained 10% animal serum in tris-buffered saline/0.09% ProClinTM 950) (Leica Microsystems) and then washed at room temperature in 1 X concentration Bond Wash solution (2 changes, 2 min each). The slides were washed in distilled water and incubated in room temperature peroxide block (3% hydrogen peroxide). The sections were washed with 1 X concentration Bond Wash solution (3 changes). Before use, the BOND III mixed the 3,3’-diamonobenzidine tetrahydrochloride (chromogen) and 0.05% of hydrogen peroxide from the refine kit to produce DAB. The sections were incubated in DAB for 10 min at room temperature then washed at room temperature in distilled water (3 changes). To intensify the DAB staining, the slides were incubated in BOND DAB enhancer (Leica Microsystems) containing copper sulfate with surfactant for 5 min at room temperature, then washed in 1 X BOND wash (3 changes at room temperature). The slides were incubated in hematoxylin for 8 min at room temperature, washed in distilled water and then with 1 X concentration BOND Wash solution. The slides were incubated in bluing reagent for 30 s at room temperature, then washed in 1 X concentration BOND Wash solution (3 changes).

Upon removal from the BOND III Immunohistochemistry Autostainer, the slides were dehydrated at room temperature in 95% reagent alcohol (3 changes, 20 s each), 100% reagent alcohol (3 changes for 20 s each), xylene (2 changes, 20 s each), and xylene (1 change for 1 min) and cover-slipped with Leica Surgipath MM24 mounting medium.

### Statistical methods

UPCR was calculated before and after treatment with abatacept by the paired Student’s *t*-test using GraphPad (http://www.graphpad.com).

## Results

Twelve patients developed nephrotic range proteinuria after KT (9 pediatric KTRs with rFSGS and 3 adult KTRs with de novo FSGS; Table 1, Fig. 1). The timing of the recurrent proteinuria was variable: there were five instances of immediate recurrence, four within 30 to 90 days post-KT, and three that occurred 6 to 8 years following KT.

Seven patients who did not respond to conventional treatment (cases 1–7) were found to be positive for podocyte B7-1 expression. These patients were treated with abatacept with subsequent improvement (2 patients, cases 5 and 6) or resolution (5 patients, cases 1–4 and 7) of proteinuria. Two pediatric patients with primary FSGS and recurrent proteinuria who did not undergo a KT biopsy responded to abatacept (cases 8 and 9) with resolution of proteinuria.

For the nine KTRs who responded to abatacept (cases 1–9), pre-treatment UPCR was 27.0 ± 20.4 (median 13, range 8–56); follow-up UPCR was 0.8 ± 1.3 (median 0.2, range 0.07–3.9), *p* < 0.004. Two KTRs underwent multiple KT biopsies over the course of 1 year, all of which were B7-1 negative, and did not respond to treatment with abatacept (cases 10 and 11). One patient developed recurrent proteinuria while receiving belatacept (case 12), later stained positive for B7-1, and subsequently did not resolve after abatacept treatment.

**Fig. 3:**
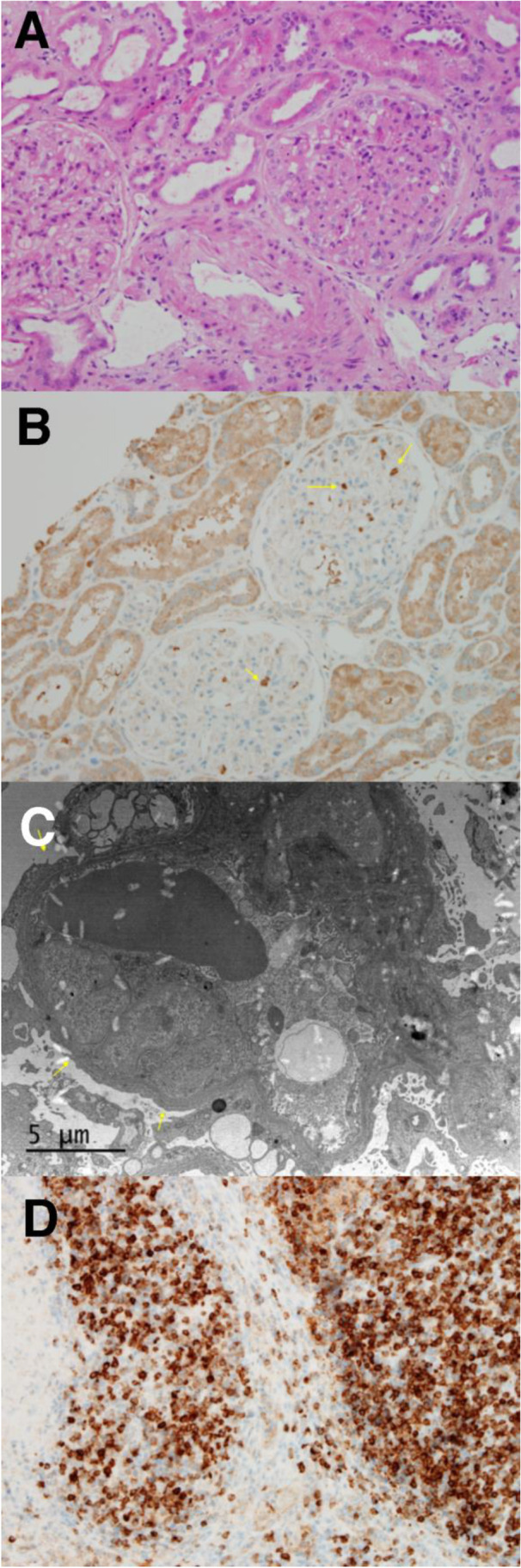
H&E, B7-1, and EM for B7-1 positive recurrence (case 6). **A** H&E (200 ×) demonstrated mesangial proliferative changes in glomeruli consistent with early segmental sclerosis. **B** Immunohistochemistry to B7-1 (200 ×) appears focally positive in podocytes (arrow) with 3 + intensity. **C** The overlying podocyte foot processes show moderate fusion (arrow) (EM, 3,500 ×). **D** Immunohistochemistry to B7-1 (200 ×) appears positive in lymph node

Two representative figures of B7-1 staining are shown: one demonstrating a kidney transplant biopsy which was positive for B7-1, as well as a positive lymph node control (Figs. 3B and D, respectively); and the other a kidney transplant biopsy that was negative for B7-1 (Fig. 5B).

### Positive cases that responded to abatacept

#### Case 1

This 22-year-old AA male presented at age two with NS secondary to FSGS. He was initially steroid responsive but became a frequent relapser. He was treated with tacrolimus and was in remission for approximately 2 years. After tacrolimus was stopped, he relapsed, became steroid resistant, and did not respond to tacrolimus when it was re-introduced. He had poor response to other immunosuppressive therapies such as cyclosporine and mycophenolate mofetil and ultimately developed kidney failure at the age of 14 years. Kidney biopsy done at 11 years of age, revealed minimal change disease and in a subsequent biopsy progressed to FSGS. He started dialysis at age 15 and underwent a deceased donor (DD) KT 15 months later. He presented with prostatitis 6 years later and was found to have nephrotic range proteinuria. UPCR peaked at 13, with serum albumin 1.6 g/dl, and creatinine 1.20 mg/dl. He was treated with five sessions of plasmapheresis and underwent a kidney transplant biopsy which demonstrated rFSGS (case 1, Table 1). The B7-1 stain on podocytes was positive (1 + intensity), with moderate fusion of podocyte foot processes (EM). He received a dose of rituximab (375 mg/m^2^) with no improvement in proteinuria. In fact, the UPCR continued to increase over the next 2 weeks, so he was treated with abatacept (10 mg/kg, intravenously, 2 doses). His UPCR fell almost immediately after the first dose of abatacept. Proteinuria resolved 1 month later (UPCR—0.17, serum albumin 4.7 g/dl, serum creatinine 1.1 mg/dl). (Fig. 2A).

#### Case 2

An 8-year-old Caucasian male with biopsy-demonstrated FSGS underwent LRD KT at the University of Florida. He received thymoglobulin induction therapy and later received a single dose of cyclophosphamide. He experienced recurrent proteinuria (peak UPCR > 50) 2 days post-transplant (case 2, Table 1). A KT biopsy showed podocyte effacement by EM and stained positive for B7-1 with co-staining for the podocyte marker synaptopodin, confirming the location of B7-1 on the podocyte. He underwent plasmapheresis and steroid treatment initially. He then received one dose of abatacept (10 mg/kg IV), underwent six more sessions of plasmapheresis, and experienced resolution of proteinuria (Fig. 2B). This was reported as patient #3 (Table 1) [36].

#### Case 3

A 34-year-old AA female with kidney failure of unknown etiology received a pediatric-en-bloc DD KT. Proteinuria developed immediately post-transplantation (peak UPCR—8) that persisted for 2 months (case 3, Table 1). A KT biopsy demonstrated de novo FSGS and stained positive for B7-1 (2 + intensity). Minimal fusion of the podocyte foot processes (EM) was noted. She was treated with a dose of rituximab (375 mg/m^2^) and three sessions of plasmapheresis. The UPCR increased from 3 to 4 over the next 30 days, and 1 month later she received abatacept (four weekly doses of 250 mg, subcutaneously) followed by an immediate fall and ultimate resolution of proteinuria (Fig. 2C). Twenty-seven months post-transplantation, creatinine was 0.75 mg/dl, with serum albumin 4.6 g/dl, and undetectable urine protein.

#### Case 4

A 25-year-old Hispanic male was diagnosed with steroid-resistant nephrotic syndrome at the age of two. At age 8, he immigrated to the USA from South America, and a kidney biopsy revealed the diagnosis of FSGS. He was treated with cyclosporine and angiotensin blockers, but developed progressive kidney dysfunction and underwent a DD KT and bilateral native nephrectomies at the age of fourteen. He experienced early rFSGS and received rituximab, and plasmapheresis with resolution of proteinuria. Eight years later, he developed kidney dysfunction and proteinuria associated with biopsy-proven antibody-mediated rejection and received bortezomib. Proteinuria persisted and he underwent a second biopsy 6 months later which again showed antibody-mediated rejection, class 2, C4d negative. This biopsy was B7-1 positive (2 + intensity). Abatacept therapy was considered, but not given and he was again treated with bortezomib and thymoglobulin (total dose 7.5 mg/kg) and rituximab. Over the next 9 months, he developed progressive proteinuria (peak UPCR—9) (case 4, Table 1) and underwent a third biopsy that again demonstrated B7-1 (still at 2 + intensity). This biopsy showed moderate fusion of podocyte foot processes (EM). He was then treated with abatacept (one dose, 10 mg/kg, intravenously), with immediate fall and resolution of proteinuria within 1 month (Fig. 2D). He returned to hemodialysis 11 years post-transplantation, 18 months after abatacept treatment, due to progressive chronic rejection.

#### Case 5

This 23-year-old AA male was diagnosed with SRNS with histologic features of FSGS at the age of eight. Treatment with mycophenolate mofetil was attempted due to steroid resistance and decreased kidney function. However, he rapidly progressed to kidney failure requiring dialysis at the age of 10. He underwent bilateral native nephrectomies and a DD KT the following year. He experienced immediate rFSGS. After multiple plasmapheresis, he was discharged with persistent NS and returned to dialysis the following year. Ten years later, he received a second DD KT (case 5, Table 1). In addition to standard induction therapy, he received plasmapheresis as well as IVIg therapy. He developed post-transplant proteinuria 45 days later and underwent plasmapheresis. A KT biopsy demonstrated rFSGS (H&E), with mild fusion of podocyte foot processes (EM) and was positive for B7-1 (2 + intensity). Eight months later, with persistent proteinuria (peak UPCR—12), he received abatacept (four doses, 250 mg/dose, subcutaneously) and experienced improvement (UPCR—2.0; serum creatinine 0.8 mg/dl) (Fig. 2E) that was sustained over 3 years.

#### Case 6

A 20-year-old male from China was diagnosed with SRNS at age 13. A kidney biopsy demonstrated collapsing variant of FSGS, and he began dialysis after 2 years. He underwent an LRD KT from his mother 6 years later and experienced severe early recurrence (UPCR > 50) (case 6, Table 1). He received plasmapheresis, steroids, IVIg, and rituximab. He underwent pre- and post-reperfusion biopsies that showed mild acute tubular injury (H&E), mild foot process focal fusion (EM), and both were negative for B7-1. He underwent another KT biopsy 10 days later that showed no significant glomerular alterations (H&E), minimal focal podocyte fusion (EM), and was positive for B7-1 (2 + intensity). He began treatment with subcutaneous abatacept and received a second dose of rituximab. After 4 months and 42 sessions of plasmapheresis, he continued to experience severe proteinuria (UPCR—26) and underwent a third biopsy demonstrating mesangial proliferative changes in the glomeruli, consistent with rFSGS (H&E), moderate overlying podocyte foot process fusion (EM), and increased intensity of B7-1 expression (3 + intensity) (Fig. 3A–C), with lymph node B7-1 staining for positive control (Fig. 3D). Plasmapheresis was stopped, and he received intravenous abatacept and high-dose steroids. Abatacept (10 mg/kg, intravenously) was continued approximately every 2 weeks for 6 months. UPCR improved to between 2 and 3.9 (Fig. 2F). Twenty-seven months later, his creatinine was 1.50 mg/dl, with UPCR—3.9, and serum albumin 4.2 g/dl (Table 1).

#### Case 7

This 22-year-old Caucasian male had a history of SRNS diagnosed at age four. Initial kidney biopsy revealed minimal change. He was treated with various immunosuppressive therapies including cyclosporine, tacrolimus, and mycophenolate mofetil. A second kidney biopsy at the age of 9 years revealed progression to FSGS and ultimately, he developed kidney failure at the age of 13 years. He underwent bilateral nephrectomies which confirmed the diagnosis of FSGS, and 2 months later, received an LRD KT from his mother at the age of 14 (patient #3 from our previously published series [26]). He experienced immediate, severe recurrence of proteinuria (UPCR—80), and stained positive for B7-1 on the post-reperfusion, but not the pre-reperfusion biopsy. The podocyte location of B7-1 was confirmed by co-staining with synaptopodin. He was treated with two doses of abatacept (IV) and proteinuria resolved [26] (Fig. 2G).

Eight years post-transplantation, he experienced an episode of severe gastroenteritis with profuse diarrhea and presented with elevated creatinine. He was rehydrated and noted to have significant proteinuria (UPCR > 10), and a low serum albumin, 2.4 g/dl (case 7, Table 1). Because his previous biopsy demonstrated B7-1, and he responded to abatacept, he was treated again, this time empirically, with abatacept. He received four doses of abatacept (10 mg/kg, IV), with resolution of proteinuria. Seven months later, his creatinine was 1.3 mg/dl, with serum albumin 4.2 g/dl, and UPCR—0.10 (Fig. 2H).

### Cases without KT biopsies that responded to abatacept

#### Case 8

**Fig. 4:**
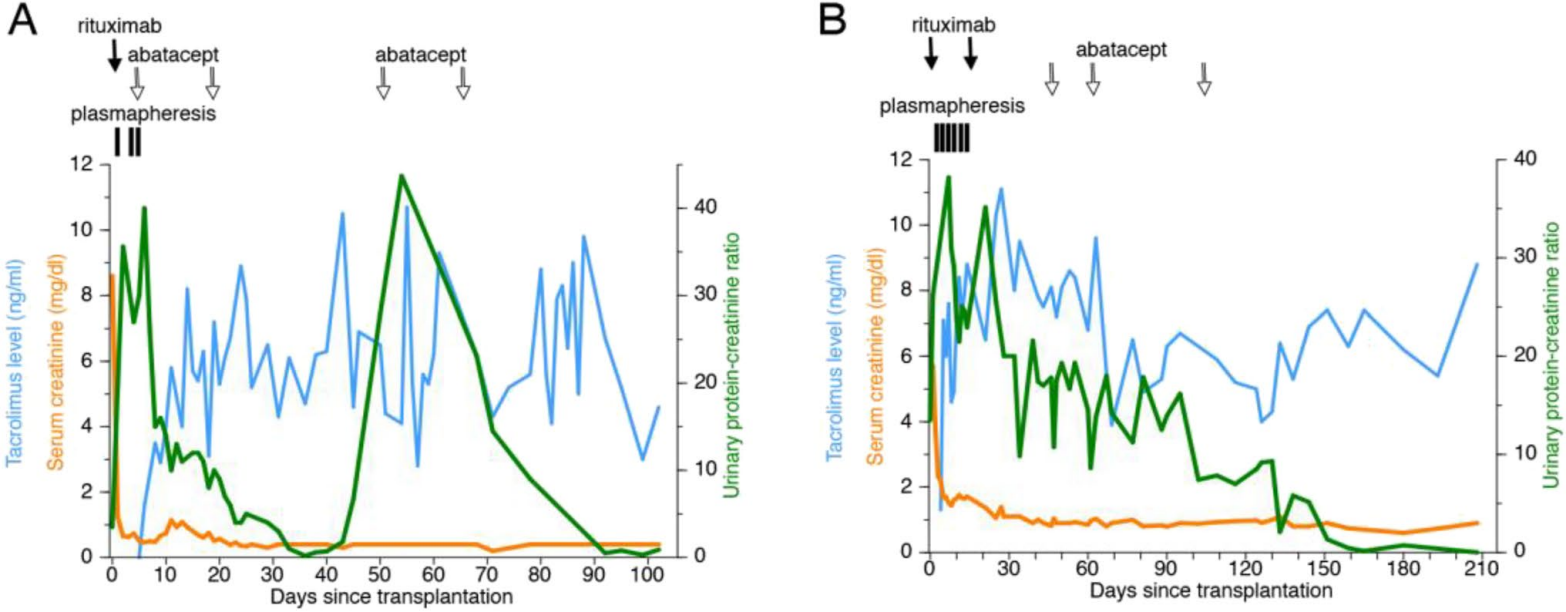
Cases without KT biopsies that responded to abatacept. Each graph shows post-transplant serum creatinine levels, tacrolimus levels, and urine protein/creatinine ratio. **A** Case 8. **B** Case 9. Also shown: rituximab infusion time(s), plasmapheresis episodes, steroids, and abatacept treatment

A 7-year-old presented with NS at the age of two. Initial kidney biopsy was consistent with minimal change and a subsequent biopsy done because of steroid resistance revealed FSGS. He was treated with multiple immunosuppressive therapies, including calcineurin inhibitors and mycophenolate mofetil. He progressed to kidney failure (biopsy-proven FSGS) by the age of 6. This patient (patient #4 from our previously published series [26]) received a DD KT the following year and experienced early recurrence (UPCR > 40). He received one dose of rituximab (375 mg/m^2^) and experienced an increase in UPCR from 28 to 38. Out of concern for plasmapheresis-related worsening anemia and the family’s refusal of transfusion due to Jehovah’s Witness beliefs, he underwent only three sessions of plasmapheresis. Since he was experiencing worsening proteinuria, he was treated with abatacept (10 mg/kg IV, two doses), with resolution of proteinuria [26]. The following month, the patient was again noted to become severely proteinuric (UPCR > 40) (case 8, Table 1). KT biopsy was considered, but again, due to religious concerns, this was not performed. The patient received two more doses of abatacept (10 mg/kg IV) and the proteinuria resolved (Fig. 4A). He has not experienced further recurrence of proteinuria. Eight years later, the patient’s creatinine is 1.10 mg/dl, UPCR < 0.07, and serum albumin 4.9 g/dl.

#### Case 9

This AA male was diagnosed with NS at age 4. He was initially a steroid responder but subsequently became steroid resistant. He was treated with multiple immunosuppressive therapies including calcineurin inhibitors, mycophenolate mofetil, and rituximab with poor response. He progressed to kidney failure at age 8. He received a DD KT at age 13 and experienced immediate recurrence of proteinuria (UPCR > 35) (case 9, Table 1). He received one dose of rituximab peri-operatively, and then a subsequent dose (375 mg/m^2^ IV) 2 weeks later. He underwent plasmapheresis on six occasions, without improvement in proteinuria. The UPCR reached a peak of 20 over the next month, and so he was treated with three doses of abatacept (10 mg/kg IV) with resolution of proteinuria (Fig. 4B). Nearly 5 years post-transplant, he has not experienced further recurrence of proteinuria. His creatinine is 1.45 mg/dl, UPCR—0.08, and serum albumin 5.1 g/dl.

### B7-1 negative cases that did not respond to abatacept

#### Case 10

**Fig. 5:**
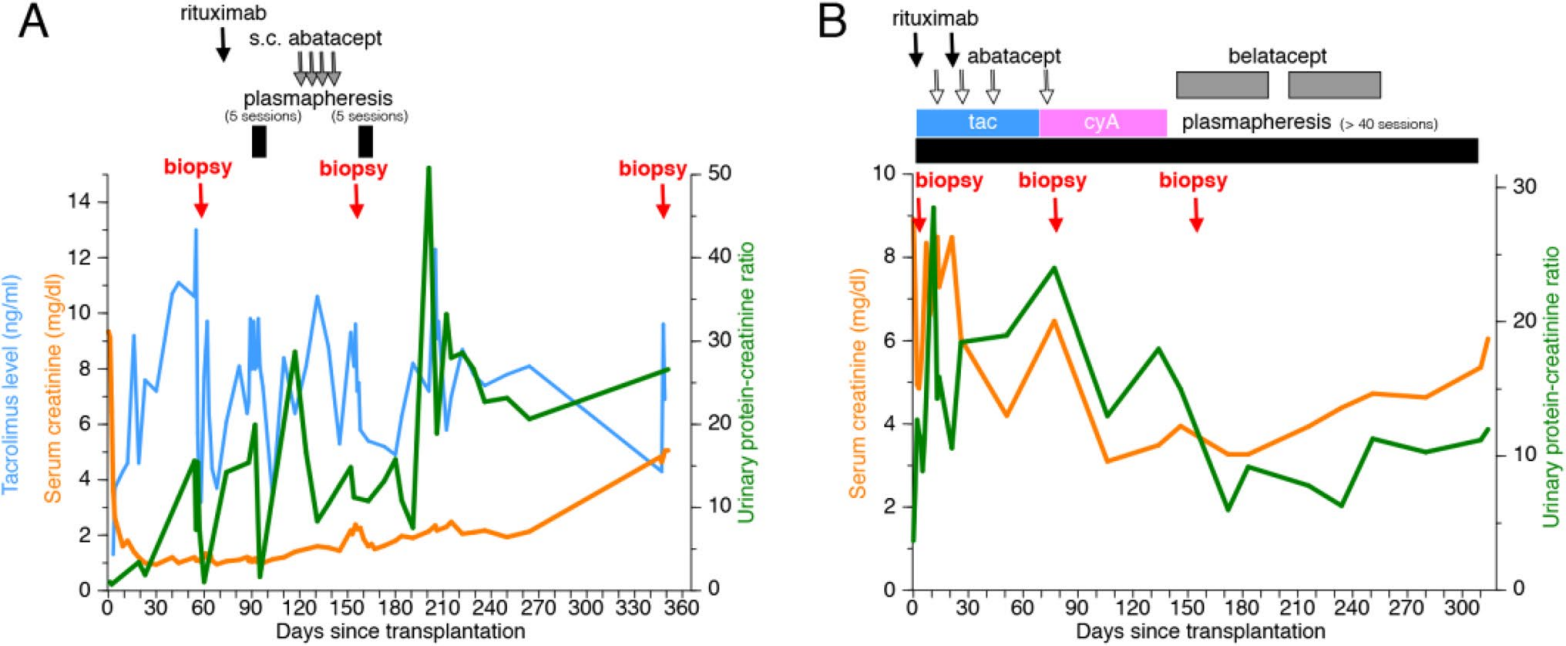
B7-1 negative cases of KT recipients with recurrent FSGS that did not respond to abatacept. **A** Case 10. **B** Case 11. Each graph shows post-transplant serum creatinine levels, tacrolimus levels (case 10 only), and urine protein/creatinine ratio. Also shown: biopsies, rituximab infusion time(s), plasmapheresis episodes, and abatacept treatment. Cyclosporine A and belatacept treatment are shown for case 11

A 48-year-old AA female with a history of ADPKD reached kidney failure and started hemodialysis. Her medical history included type 2 diabetes mellitus and hypertension. Eight years later, she underwent DD KT. She experienced nephrotic range proteinuria (UPCR > 10) within 1 month of transplantation and was found to have de novo FSGS on biopsy (case 10, Table 1). This biopsy was negative for B7-1. She was treated with rituximab and plasmapheresis without improvement in proteinuria. Due to her lack of response to available therapy, she received a course of abatacept (four weekly doses of 250 mg, subcutaneously) without improvement of proteinuria. Over the course of the next 10 months, she experienced persistent proteinuria (peak UPCR—50) and underwent two more KT biopsies, which showed FSGS with mild foot process fusion (EM). Both biopsies remained B7-1 negative. Her proteinuria did not resolve (Fig. 5A). One year later, creatinine was 4.82 mg/dl, with UPCR—26.7, serum albumin 2.3 g/dl, and she resumed dialysis.

#### Case 11

**Fig. 6:**
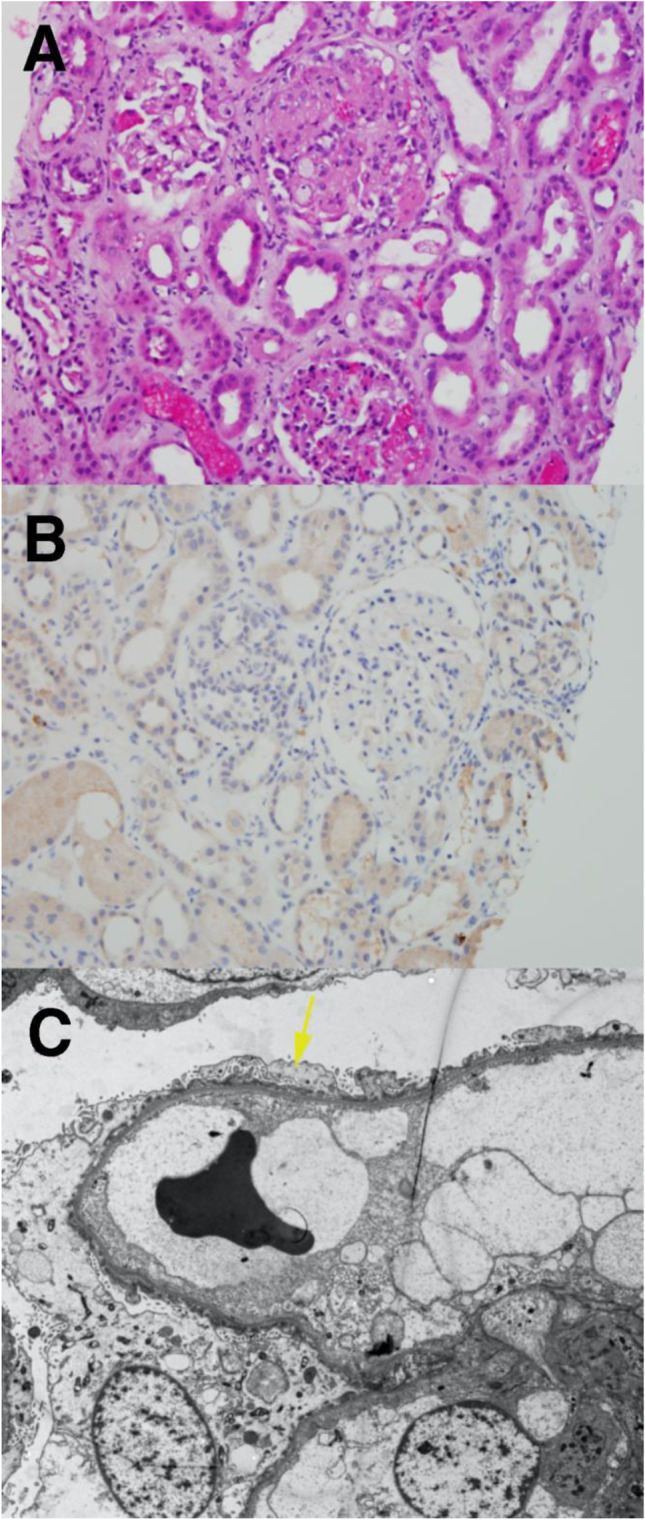
H&E, B7-1, and EM in B7-1 negative recurrence (case 11). **A** H&E (200 ×) demonstrated focal segmental glomerulosclerosis, with frequent non-collapsing segmental sclerosis, approximately 13 of 25 glomeruli. **B** Immunohistochemistry to B7-1 (200 ×) appears negative in podocytes. **C** The overlying podocyte foot processes show moderate fusion (arrow) (EM, 3740 ×)

This 23-year-old Caucasian male, with a history of biopsy-proven FSGS, at the age of 20, progressed rapidly to dialysis 4 months later. He received an LRD KT from his mother 7 months after starting dialysis. He experienced severe recurrence of proteinuria (UPCR—28) 48 h post-transplant (case 11, Table 1). He was treated with a course of plasmapheresis initially. He developed delayed graft function and experienced septicemia, fluid overload, hypertension, and *Clostridioides difficile*-related diarrhea. He required hemodialysis during the episode of sepsis. He underwent three kidney transplant biopsies: intra-operative, 3 months, and 5 months after the transplant. The first biopsy, pre- and post-reperfusion, obtained in the operating room, showed focal mild tubulo-interstitial injury (H&E), with no foot process effacement (EM), and B7-1 was negative (pre- and post-reperfusion). The second and third biopsies (Fig. 6A–C) showed FSGS (H&E), with moderate fusion of overlying foot processes (EM), and both remained B7-1 negative. His treatment included multiple courses of plasmapheresis, rituximab, belatacept, and four doses of abatacept (500 mg IV, each). Eleven months after KT, despite months of plasmapheresis, he returned to dialysis (Fig. 5B).

### B7-1 positive case that received belatacept and did not respond to abatacept

#### Case 12

**Fig. 7:**
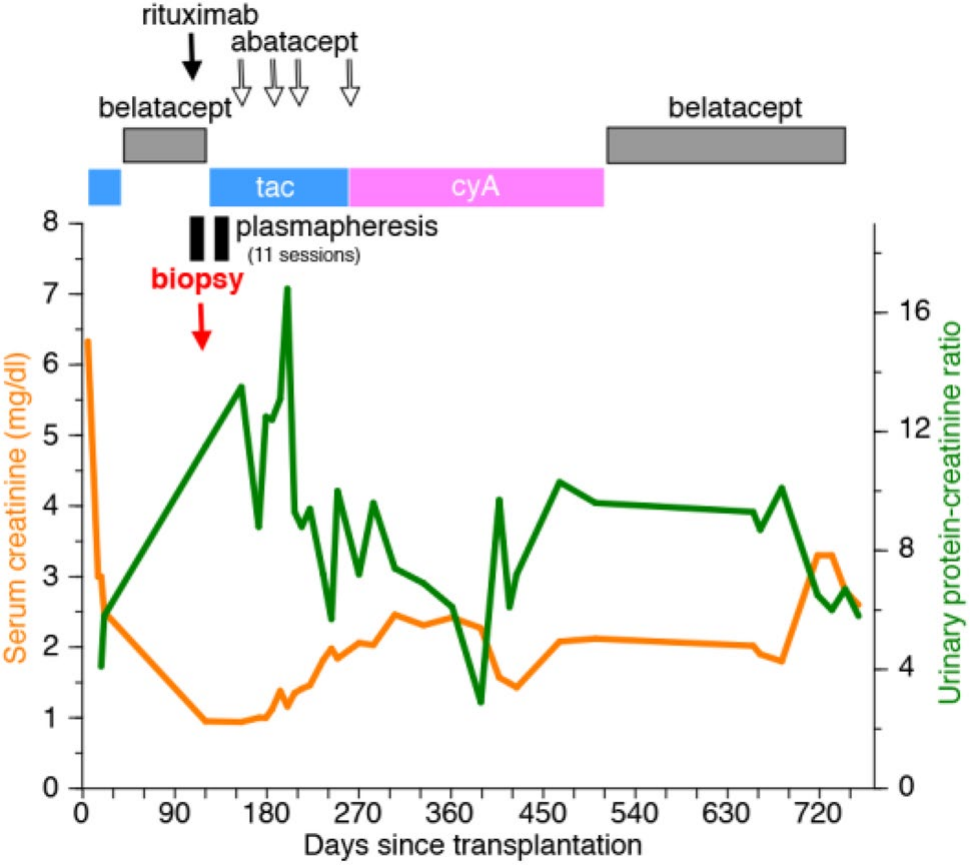
B7-1 positive case that did not respond to abatacept. The graph (case 12) shows post-transplant serum creatinine levels, tacrolimus levels, and urine protein/creatinine ratio. Also shown: rituximab infusion time(s), plasmapheresis episodes, steroids, belatacept, and abatacept treatment

This 78-year-old Caucasian female with kidney failure secondary to type 2 diabetes and hypertension underwent a native kidney biopsy that demonstrated CKD 5. She was on hemodialysis for 1 year and received a DD KT (case 12, Table 1). She experienced immediate graft function but developed tacrolimus-related neurotoxicity and was switched to belatacept 1 month after KT. She developed nephrotic range proteinuria 3 months after transplantation. A KT biopsy 1 month later demonstrated de novo FSGS, with podocyte staining positive for B7-1 (2 + intensity) and moderate podocyte foot process fusion (EM). Belatacept was discontinued, and she received one dose of rituximab and eleven sessions of plasmapheresis. Her degree of proteinuria continued to increase 1 month after stopping belatacept, and she received the first of four doses of abatacept (500 mg IV, each). Proteinuria increased initially to a peak UPCR > 16, then fell to between 3 and 4. However, over the next year proteinuria increased to a peak of 10. Belatacept was restarted to avoid calcineurin inhibitor toxicity. Twenty-three months post-transplant, her creatinine was 3.60 mg/dl, UPCR—6, and serum albumin of 2.7 g/dl (Fig. 7) with unresolved FSGS.

## Discussion

We describe nine KTRs with post-transplant FSGS, who failed conventional therapy including rituximab, plasmapheresis, steroids, and calcineurin inhibitors [11–13]. These patients received abatacept with subsequent improvement or resolution of proteinuria and stabilization of kidney function. The resolution for the second time (cases 7, 8), without plasmapheresis or rituximab, strongly supports the beneficial effect of abatacept. The other patients in the University of Miami series (cases 1, 3–6 and 9) experienced rising levels of proteinuria after rituximab and plasmapheresis therapy, providing evidence of failure of conventional therapy, leading to the clinical decision to treat with abatacept. Case 2 from the University of Florida team received a different protocol including thymoglobulin induction and cyclophosphamide, with plasmapheresis. After treatment with abatacept, his proteinuria resolved. Together these cases support the role of B7-1 staining in KT biopsies, and treatment with abatacept for those with B7-1 positive biopsies in treatment-resistant post-transplant FSGS.

Two patients (cases 1 and 9) were initially steroid-sensitive, developing steroid resistance over time, and were therefore at particularly high risk for experiencing recurrent proteinuria after KT [37]. The time from transplant to development of nephrotic range proteinuria varied from immediately up to 8 years following KT. Two patients (cases 4 and 6) demonstrated B7-1 on more than one KT biopsy, separated by months, and responded to abatacept. One other patient (case 5) continued with proteinuria associated with B7-1 positive rFSGS and responded promptly to abatacept following treatment 6 months later. Overall, there was a significant decline in UPCR in this mostly pediatric, B7-1 + cohort with post-transplant FSGS. In two cases (cases 10 and 11), where B7-1 staining was negative on each of three biopsies during the first year after transplant, there was no improvement in the degree of proteinuria after treatment with abatacept, resulting in graft loss. In two cases (2 and 7) of KT biopsies, B7-1 staining was colocalized with synaptopodin, confirming the location of B7-1 on the podocyte.

There have been reports after our initial publication [26] that either (1) could not identify B7-1 on KT biopsies [28–31], or (2) did not demonstrate a response to CTLA-4Ig, including abatacept and belatacept in rFSGS [28, 32, 33]. Although there has been controversy regarding tissue podocyte B7-1 staining [30, 31, 38], the reproducibility is apparent, given staining of positive controls (lymph nodes) by others [28] and by our group (Fig. 3D). Moreover, podocyte B7-1 staining has been demonstrated in our two previous reports [26, 36] and currently in our post-transplant FSGS patients, of which Fig. 3C is an example, and by others [32] using commercially available antibodies. Differences in technical aspects, for example, frozen vs. fresh kidney tissue samples (although this has been disputed [39]), monoclonal vs. polyclonal anti-B7-1 antibody staining, and different anti-species secondary antibody counter-staining, may have also played a role in others not being able to identify podocyte B7-1 [28–31, 33, 39].

Patient age differences between our work and those of other centers [28, 31, 33], perhaps also contributed to the lack of positive B7-1 staining on KT biopsies (see Table 2). Our patients were from a predominantly pediatric age group. In contradistinction, 7/9 of the rFSGS patients from one cohort [28] and all ten patients in the other two [32, 33] were adults. Adults with primary podocytopathy are much less likely to be positive for B7-1 [40]. Two of these groups [28, 32] reported that their patients with rFSGS did not respond to abatacept (see Table 2). However, all five of the patients in one cohort were B7-1 negative [28]. The other group [32] included only one patient (case 1 in their series) treated with abatacept, whose B7-1 staining was also negative [41]. Our work is consistent with these results, since our B7-1 negative KT recipients (cases 10 and 11) did not respond to abatacept. Table 2 shows the lack of clinical response to abatacept in the absence of B7-1 positive staining in our patients as well as those in the literature (0/9).

**Table 2:** Response of patients with post-KT FSGS to abatacept or belatacept in context of B7-1 staining

Authors and references	Response to abatacept			Response to belatacept		Biopsy preparation	Patient age (years)
	No biopsy	Biopsy					
		B7-1 pos	B7-1 neg	B7-1 pos	B7-1 neg		
Burke et al. current	2/2	7/7 0/1^1^	0/2^2^	0/1^1^	0/1^2^	Fresh (see text)	2, 2, 4, 4, 6, 8, 8, 13, 20, 34, 56, 78
Delville et al. [28]			0/5		0/4	Fixed in ethanol-formalin-acetic acid/paraffin	5, 12, 22, 30, 36, 49, 49, 54, 56
Alachkar et al. [32]			0/1	0/4^3^		Frozen	26, 36, 37, 50, 57
Grellier et al. [33]					0/5	Not stated	24, 39, 50, 51, 63
Garin et al. [36]		1/1^4^	0/1	0/1		Frozen	8, 21, 44
Total	9/10		0/9	0/16			

^1^Case 12 in Burke et al. treated with belatacept first, then abatacept (see text and Fig. 7)

^2^Case 11 in Burke et al. treated with abatacept first, then belatacept (see text and Fig. 5)

^3^Variable staining and diagnosis

^4^Garin et al. patient 3 (same as Burke et al. case 2)

Furthermore, in none of the three reports [28, 32, 33] was there a response to belatacept (see Table 2). Similar to our experience (case 12), one reported patient (patient #9) developed proteinuria while receiving belatacept [28], confirming that belatacept is not effective in preventing proteinuria in rFSGS. However, unlike their patient, our patient’s KT biopsy stained positive for B7-1. Our patient’s proteinuria subsequently did not resolve after abatacept treatment. Abatacept, the first generation anti-CTLA-4Ig, binds to B7-1 with greater avidity than B7-2, and belatacept binds to both, with preferential binding of B7-2 [42]. Podocytes express B7-1, but not B7-2 [24]. Therefore, abatacept is likely the better agent in this clinical context [41]. In addition, a second patient (case 11) in our series did not respond to belatacept, and a patient with rFSGS whose KT biopsy was B7-1 positive did not respond to belatacept (case 5 [36]), supporting the lack of efficacy of belatacept reported by others [28, 32, 33]. Table 2 details the lack of any beneficial response to belatacept in our series and in the published literature, regardless of B7-1 staining (0/16).

Our twelve patients with post-KT FSGS are a heterogeneous group with (1) nine cases of rFSGS (< age 21) and three cases of de novo FSGS (adults); (2) racial mix including 5 African Americans, 4 Caucasians, and 3 Hispanics; and (3) a range of timing of nephrotic range proteinuria—most of which were immediately after KT, but others as long as 8 years after KT. Nonetheless, the common features within this diverse group of post-KT FSGS are B7-1 staining on KT biopsies and clinical response to abatacept. We also show the lack of response to abatacept in the absence of B7-1 staining and the absence of response to belatacept. The single non-response to abatacept in the context of positive B7-1 staining was possibly influenced by prior treatment with belatacept. For those patients experiencing rFSGS who are B7-1 negative, there may be other mechanistic influences perpetuating proteinuria that we hope will be elucidated in future studies.

This study is a compilation of our most recent experience, in which B7-1 staining was associated with a favorable response to abatacept. Limitations include the following: (1) B7-1 staining is now a routine test at our center; however, colocalization with synaptopodin is not routinely performed, and (2) with the exception of cases 7 and 8, where abatacept was used successfully without other treatment, the other cases have been in the context of multi-agent approaches, which, while deemed to be ineffective, could potentially confound the role of abatacept. We hope that this report will lead to renewed study of mechanisms associated with podocyte B7-1 expression and response to abatacept treatment in proteinuric kidney disease [43].

## Conclusion

It is important to emphasize that reversing post-transplant FSGS resistant to conventional therapy is critically important, particularly in the high-risk pediatric population, since the unsuccessful treatment of recurrence almost invariably leads to poor outcomes, as reported here (cases 10–12) and by others [9, 13, 28, 32, 33, 44]. Our experience suggests that the presence of B7-1 on podocytes in KT biopsies of recipients with post-transplant FSGS identifies a subset of patients who may benefit from treatment with abatacept.

## Supplementary Information

Below is the link to the electronic supplementary material.

Graphical Abstract (PPTX 74.2 KB)

## Abbreviations

AA: African American
ACEI: Angiotensin converting enzyme inhibitor
ARB: Angiotensin receptor blocker
DD: Deceased donor
EM: Electron microscopy
FSGS: Focal segmental glomerulosclerosis
H&E: Hematoxylin and eosin
IVIg: Intravenous immunoglobulin
KT: Kidney transplant
KTR: Kidney transplant recipient
LD: Living donor
MCD: Minimal change disease
NS: Nephrotic syndrome
PP: Plasmapheresis
rFSGS: Recurrent focal segmental glomerulosclerosis
SRNS: Steroid-resistant nephrotic syndrome
UPCR: Urine protein/creatinine ratio
